# Zuo Jin Wan reverses P-gp-mediated drug-resistance by inhibiting activation of the PI3K/Akt/NF-κB pathway

**DOI:** 10.1186/1472-6882-14-279

**Published:** 2014-08-01

**Authors:** Hua Sui, Shu-Fang Pan, Yu Feng, Bao-Hui Jin, Xuan Liu, Li-Hong Zhou, Feng-Gang Hou, Wen-Hai Wang, Xiao-Ling Fu, Zhi-Fen Han, Jian-Lin Ren, Xiao-Lan Shi, Hui-Rong Zhu, Qi Li

**Affiliations:** Department of Medical Oncology, Shuguang Hospital, Shanghai University of Traditional Chinese Medicine, Shanghai, 201203 China; Interventional Cancer Institute of Integrative Medicine, Shanghai University of Traditional Chinese Medicine, Shanghai, 201203 China; Department of General Surgery, Shuguang Hospital, Shanghai University of Traditional Chinese Medicine, Shanghai, 201203 China; Department of Oncology, Shanghai Municipal Hospital of Traditional Chinese Medicine, Shanghai, 200071 China

**Keywords:** Drug-resistance, *Akt*, *NF-κB*, *ABCB1*, *ZJW*

## Abstract

**Background:**

Zuo-Jin-Wan (ZJW), a traditional Chinese medicine formula, has been identified to be effective against drug resistance in cancer. In the present study, we investigated the effect of ZJW on acquired oxaliplatin-resistant and the PI3K/Akt/NF-κB pathway *in vitro*.

**Methods:**

We tested the dose–response relationship of ZJW on reversing drug-resistance by CCK-8 assay and flow cytometry analysis *in vitro*. The protein expression of P-gp, MRP-2, LRP, and ABCB1 mRNA expression level were evaluated by Western blot and quantitative RT-PCR. The activities of PI3K/Akt/NF-κB pathway were also examined with or without ZJW, including Akt, IκB, p65 and their phosphorylation expression.

**Results:**

We found that ZJW significantly enhanced the sensitivity of chemotherapeutic drugs and increased oxaliplatin (L-OHP)-induced cell apoptosis in a time- and dose-dependent manner. Moreover, both ZJW and a PI3K specific inhibitor (LY294002) suppressed phosphorylation of Akt (Ser473) and NF-κB, which is necessary in the activation of the PI3K/Akt/NF-κB pathway. The effect of ZJW in reversing drug-resistance and suppressing phosphorylation of Akt (Ser473) and NF-κB were weakened after treatment with a PI3K/Akt activator in HCT116/L-OHP cells.

**Conclusions:**

Our study has provided the first direct evidence that ZJW reverses drug-resistance in human colorectal cancer by blocking the PI3K/Akt/NF-κB signaling pathway, and could be considered as a useful drug for cancer therapy.

## Background

Colorectal cancer (CRC) is the second commonest cause of cancer-related deaths in the Western World, and resistance to chemotherapy remains the primary reason for treatment failure in advanced CRC [1]. Chemotherapy is an important therapeutic method for CRC patients. However, the development of drug resistance usually results in the failure of chemotherapy [2]. Drug-resistance of a tumor can be divided into targeting resistance, which arises to one or two special drugs, and multidrug resistance (MDR), which means the resistance of cancer cells to one chemotherapeutic drug accompanied by resistance to other chemotherapeutic drugs that may have different structures and mechanisms of action [3].

To date, the most important potential mechanism of drug-resistance is the ABC transporter family, including the well known P-glycoprotein (P-gp, encoded by the ABCB1 gene), MDR-associated protein 1 (MRP1, encoded by the ABCC1 gene) and ABC subfamily G member 2 [4]. To reverse cellular transport protein-mediated drug-resistance, studies have been conducted to explore possible mechanisms via signaling pathways, including phosphatidylinositol 3-kinase (PI3K) signal transduction.

The phosphoinositide 3-kinases (PI3Ks), a family of lipid kinases that propagate intracellular signaling cascades regulating a wide range of cellular processes, are believed to be one of the reasons for the development of chemoresistance during cancer therapy [5]. PIP3, which is a PI3K phosphorylation product, brings two PH domain-containing serine/threonine kinases, phosphoinositide-dependent kinase 1 (*PDK1*), and AKT into close proximity [6]. Previous studies have also indicated that Akt phosphorylation can induce drug-resistance in several kinds of tumors, such as ovarian cancer [7], breast cancer [8], and hepatocellular carcinomas [9]. Furthermore, AKT impedes negative regulation of the transcription factor NF-κB, leading to increased transcription of antiapoptotic and prosurvival genes [10]. In fact, spontaneous activation of NF-κB has been detected in human colorectal cancer tissues, and activation of NF-κB is believed to result in the chemoresistant phenotype in colorectal cancer cells [4, 11].

Zou-Jin-Wan (ZJW), a traditional Chinese medicine formula, has been used as a therapy to slow colorectal cancer progression, improve quality of life, and prolong survival times. ZJW is composed of *Rhizoma Coptidis* and *Fructus Evodiae* in the ratio 6:1 (w/w). *Coptis chinensis* Franch, the main component of *Rhizoma Coptidis*, has been reported to reverse drug-resistance cancer [12, 13] and suppress the nuclear translocation of p50/p65 NF-κB proteins and their binding to target genes [14]. *Fructus Evodiae* and its major alkaloid component, evodiamine, have been demonstrated to induce apoptosis in human melanoma A375-S2 cells, lead to inactivation of the PI3K/Akt/NF-κB pathway, and stop the translocation of NF-κB [15]. Although the role of *Rhizoma Coptidis* and *Fructus Evodiae* in the PI3K/Akt/NF-κB pathway have been investigated extensively, much less is known about their potential role in regulating drug-resistance when mixed in ZJW.

Although the ZJW herbal formula has anti-cancer effects, its underlying mechanism in reversing drug-resistance remains unknown. In this study, we aimed to elucidate the effect and the molecular mechanisms of the Chinese herbal formula, ZJW, in human cancer cells *in vitro.*

## Methods

### Preparation of the extracts for ZJW

Rhizoma Coptidis and Evodia were purchased from Lei-Yun-Shang Pharmaceutical Group (Shanghai) and identified as the coptis of ranunculaceae and the fruit of Tetradium, respectively. They were formally identified by Professor DZ Wu (The School of Pharmacy, Shanghai University of Traditional Chinese Medicine, Shanghai, China).

ZJW was formulated by Rhizoma Coptidis and Evodia (in a ratio of 6:1). All the herbs were purchased from ShuGuang Hospital herb store and were done as described previously [1]. Briefly, the mixture (70 g) was extracted twice for 1 h each time by refluxing in ethanol (1:8, v/v). The filtrates were concentrated and dried in vacuum at 60°C. The concentrated extract was then dried by lyophilization to obtain the ZJW extract at a yield of dried powder of 24.4%. The extract was stored at 4°C, and its preparations were standardized, regulated and quality controlled according to the guidelines defined by Chinese State Food and Drug Administration (SFDA).

### Cell culture and reagents

Human colorectal cancer HCT116 cells were purchased from the Shanghai Cell Collection (Shanghai, China). The HCT116/L-OHP MDR cell lines were established and maintained in our laboratory. Cells were grown in RPMI 1640 medium supplemented with 10% (v/v) heat-inactivated fetal calf serum, 2 mM glutamine, 100 units/mL penicillin, and 100 μg/mL streptomycin (Invitrogen, Carlsbad, CA) at 37°C in a 5% CO_2_ humidified atmosphere. HCT116/L-OHP cells were routinely maintained in a medium containing 5,000 ng/mL oxaliplatin (L-OHP). Monoclonal antibodies against P-gp, MRP-2, LRP, IκB, Akt, p65, and GAPDH were purchased from Cell Signaling Technology (Beverly, MA, USA).

### Cell viability assays

Cell proliferation was determined using the cell counting kit, CCK-8. Briefly, cells were seeded in 96-well plates at 1 × 10^4^ cells/well. When the cells reached 60% confluence, the medium was removed and replaced with fresh medium containing varying concentrations of ZJW or its mixture with anti-tumor drug (L-OHP) and incubated for 48 h. The CCK-8 assay was then performed: after 2 h of incubation with culture medium containing the CCK-8 reagent, the absorbance was read at 450 nm using a microplate assay reader (Labsystems Dragon, Wellscan). Relative inhibitory rate of cell growth was calculated according to the formula: R = (A2 − A1)/A2 × 100% and P = A1/A2 × 100%, in which R was relative inhibitory rate, P was relative proliferation ratio of cell growth, A1 is the mean absorbance of transfected cells, and A2 is the mean absorbance value of untransfected control cells without drug treatment. All experiments were done with five wells per experiment in triplicate.

### Apoptosis and cell-cycle assay

Cells were seeded in 6-well plates (4 × 10^5^ cells/well). After 12 h, three dose concentrations of ZJW were added. Flow cytometry was used to detect apoptosis by determining the relative amount of AnnexinV-FITC-positive-PI-negative cells, as previously described [1]. Unstained cells, cells stained with Annexin V-FITC alone, and cells stained with propidium iodide alone were used as controls. Singly stained cells were used to adjust electronic compensation on the FL1 and FL2 channels. After apoptosis assay, cell cycle distributions were analyzed with the ModFit program (BD, San Diego, CA, USA). All samples were assayed in triplicate, and the fraction of each cell cycle phase was calculated.

### Western blot analysis

Whole cell lysate for SDS-PAGE and western blot analysis for P-gp, MRP-2, LRP, IκB, Akt, p65, and phosphorylation of Akt and IκB expression was prepared as previously reported [1]. The lysate was incubated on ice in immunoprecipitation assay buffer for 2 h before being homogenized using a mortar and pestle. The homogenized sample was centrifuged, and the supernatant was collected and stored at −80°C. Equal loading was confirmed with GAPDH. Densitometric analysis was done using the Scion Imaging software (Scion Corporation), with GAPDH as internal control.

### Quantitative RT-PCR analysis

Tumor cells were homogenized and suspended with an RNAspin Mini Kit (GE Healthcare, Waukesha, WI, USA) for RNA isolation according to the manufacturer’s instruction. For cDNA synthesis, 1 μg of total RNA was reverse-transcribed using oligo-dT primers and the Superscript Amplification System (Life Technologies, Carlsbad, CA, USA). Quantitative RT-PCR was carried out using SYBR Green PCR Master Mix (Life Technologies). The PCR parameters consisted of initial polymerase activation at 94°C for 5 min, followed as previously described [1]. Amplification of GAPDH RNA, a relatively invariant internal reference was performed in parallel, and cDNA amounts were normalized to equivalent GAPDH mRNA levels. Oligonucleotide primers for ABCB1 and GAPDH were as follows: Oligonucleotide sequence of ABCB1 (379 bp), F: 5′-TAATGCGACAGGAGATAGG-3′, R: 5′-TGCCATTGACTGAAAGAAC-3′, Oligonucleotide sequence of GAPDH (306 bp), F: 5′-ACCCACTCCTCCACCTTTGA-3′, R: 5′-CTGTTGCTGTAGCCAAATTCGT-3′.

### ABCB1 promoter activity by vector transient transfection and dual luciferase assay

Transfection procedures were performed according to manufacturers’ instructions, with Lipofectamin 2000 as transfection reagent (Invitrogen). Briefly, 2 × 10^3^ cells were plated in each well of a 96-well plate and incubated overnight. A mixture of Lipofectamine 2000 (10 nM) with ABCB1 promoter recombinant vector pGL3-Basic-ABCB1 promoter (0.8 μg/well) was added, followed by a 48 h incubation in regular medium. Then cells were harvested and analyzed using a dual-luciferase assay kit (Shanghai Lai’an Biotech. Co., Ltd, Shanghai, China) as previously reported [1].

### Chromatin immunoprecipitation

Chromatin immunoprecipitation (ChIP) was done as described previously [16]. N-ethylmaleimide was added to the cell lysis buffer at a final concentration of 10 mM to preserve poly-ubiquitinated protein conjugates.

### Statistical analysis

All experimental data are expressed as mean ± standard deviation of at least three independent experiments performed in duplicate. Significance was determined by a one-way analysis of variance (ANOVA) and Holm’s multiple-comparison test. Statistical significance was set at a *P*-value of less than 0.05. All analyses were carried out using SPSS13.0 (SPSS, Chicago, IL, USA).

## Results

### ZJW modulates L-OHP resistance in vitro

**Figure 1:**
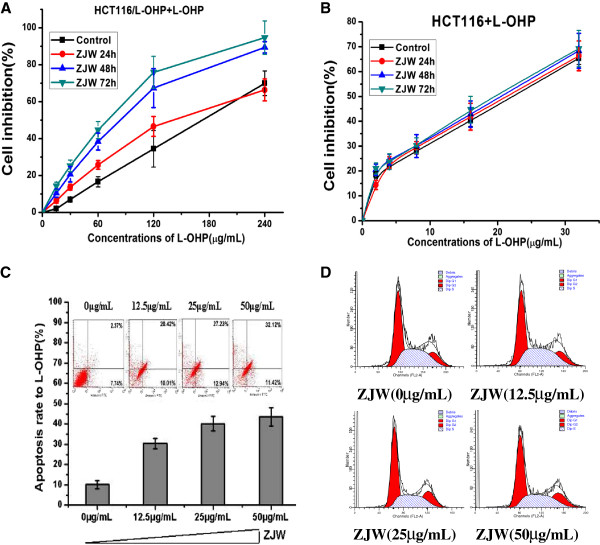
**ZJW modulates the MDR phenotype in a dose- and time-dependent manner. (A&B)** CCK-8 assay was used to detect the cellular inhibition of L-OHP in HCT116 cells and HCT116/L-OHP cells treated with ZJW at 50 μg/mL for 24, 48, and 72 h. **(C)** Flow cytometry analysis of apoptosis with Annexin V-FITC/PI binding to HCT116/L-OHP cells after treatment with ZJW at 0, 12.5, 25, and 50 μg/mL for 48 h. **(D)** Flow cytometry analysis was used to distinguish cells in different phases of the cell cycle as Methods described.

We previously showed that ZJW enhances the inhibition rate of chemotherapeutic agents in a dose-dependent manner in MDR tumor cells, including colorectal cancer cells (HCT116/L-OHP), gastric carcinoma cells (SGC7901/DDP), and hepatic carcinoma cells (Bel/Fu). To further examine the effect of ZJW in reversing chemoresistance, we detected the cell proliferation in response to chemotherapeutic agents by the CCK-8 assay and apoptosis by flow cytometry. As shown in Figure 1A, ZJW caused a significant decrease in the cell inhibition rate of chemotherapeutic agents in a time-dependent manner. However, sensitive cell proliferation treatment with ZJW and L-OHP were not found to be significantly different from L-OHP control group (Figure 1B). Similar to our previous study, ZJW increased L-OHP-induced cell apoptosis in a dose-dependent manner (Figure 1C). To further examine the effect of ZJW in cell cycle, Flow cytometric analysis was used after HCT116/L-OHP cells in response to ZJW only without HCT116/L-OHP. However, the cell cycle analyses data showed that there was no change in any phase arrest, especially in sub-G0 population of cell (Figure 1D), suggesting that ZJW did not alter cell cycle in HCT-116/L-OHP cells. Therefore, these data suggest that ZJW is responsible for reversing chemoresistance in its lowest dosage of the IC_10_ with a time- and dose-dependent manner.

### The effect of ZJW and PI3K/Akt pathway on L-OHP resistance

**Figure 2:**
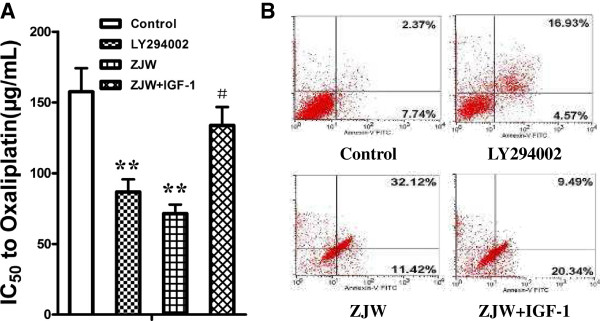
**ZJW reversed constitutive as well as PI3K/Akt pathway-induced MDR phenotype. (A)** Anti-proliferative IC_50_ values of L-OHP on HCT116/L-OHP cell treated with LY294002 (20 μM, 2 h), ZJW (50 μg/mL, 48 h), and a combination of ZJW (50 μg/mL, 48 h) and IGF-1 (100 ng/mL, 48 h), were analyzed by CCK-8. Data are presented as mean ± SD of triplicate experiments. **P < 0.01 *vs.* control, ^#^ *P* < 0.05 *vs.* ZJW group. **(B)** Flow cytometry analysis of apoptosis with Annexin V-FITC/PI binding to HCT116/L-OHP cells treated with LY294002 (20 μM, 2 h), ZJW (50 μg/mL, 48 h), and a combination of ZJW (50 μg/mL, 48 h) and IGF-1 (100 ng/mL, 48 h).

Previous studies have demonstrated that the PI3K/Akt pathway is activated in various tumor MDR cell types. To ascertain whether the reversal of drug-resistance by ZJW in colorectal cancer cells is correlated with the activation of the PI3K/Akt pathway, a PI3K specific inhibitor (LY294002) and activator (IGF-1) were added to HCT116/L-OHP cells as described. As expected, pretreatment with LY294002 or ZJW increased the sensitivity to chemotherapy and allowed the chemotherapeutic drugs to induce cell apoptosis (Figure 2A and B). However, the combination ZJW and IGF-1 weakened the reversal of L-OHP resistance as compared with the ZJW or LY294002 groups (Figure 2A and B).

### ZJW inhibits P-gp expression and the effect of the PI3K/Akt pathway

**Figure 3:**
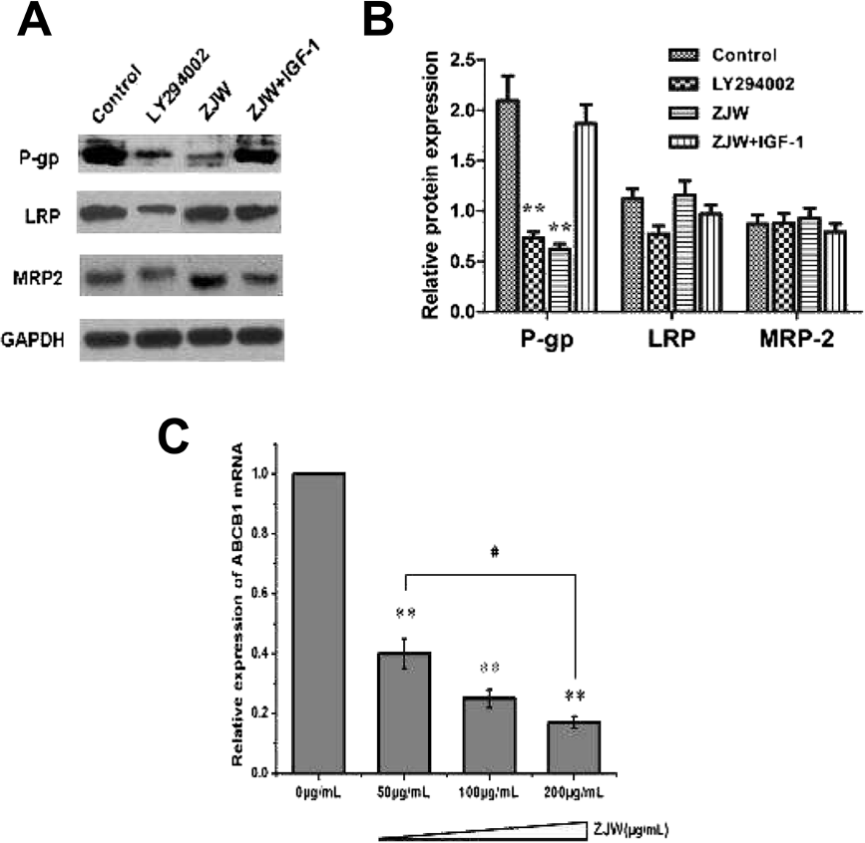
**ZJW inhibits P-gp expression and the effect of the PI3K/Akt pathway. (A)** Western blotting assay was carried out to detect the level of P-gp, LRP, and MRP-2 in HCT116/L-OHP cells treated with LY294002 (20 μM, 2 h), ZJW (50 μg/mL, 48 h), and a combination of ZJW (50 μg/mL, 48 h) and IGF-1 (100 ng/mL, 48 h). GAPDH was used to ensure equal loading of proteins in each lane. **(B)** Blots were photographed and quantitated; the data are from three independent experiments. **(C)** Real-time PCR was performed to detect ABCB1 mRNA *in vivo*. Quantification of ABCB1 mRNA was performed by assigning a value of 1 to the control group treatment with ZJW (50 μg/mL). Statistical difference was analyzed by student’s *t*-test, **P < 0.01 *vs.* control group; ^#^ *P* < 0.05 *vs.* ZJW (50 μg/mL) group. This is a representative result of three experiments with similar results.

To further investigate the mechanisms underlying the effect of ZJW and its relationship with the PI3K/Akt pathway, three cell membrane-bound ATP binding cassette (ABC) transporters, P-gp, MRP-2, and LRP, were determined by western blot. Previous study revealed that ZJW administration to MDR cells was accompanied with the downregulation of P-gp, but not other proteins [1]. As shown in Figure 3A and B, LY294002 decreased the expression of P-gp significantly. Moreover, P-gp expression was up-regulated when cells were treated with ZJW and IGF-1. Similarly, we observed a down-regulation in the expression of ABCB1 mRNA in a dose-dependent manner when HCT116/L-OHP cells were treated with ZJW (Figure 3C). These findings support the hypothesis that inhibition of P-gp by ZJW is attributable to suppression of the translational process in the activation of the PI3K/Akt pathway.

### ZJW suppresses P-gp mediated drug-resistance by inhibiting activation of the PI3K/Akt/NF-κB pathway in vitro

**Figure 4:**
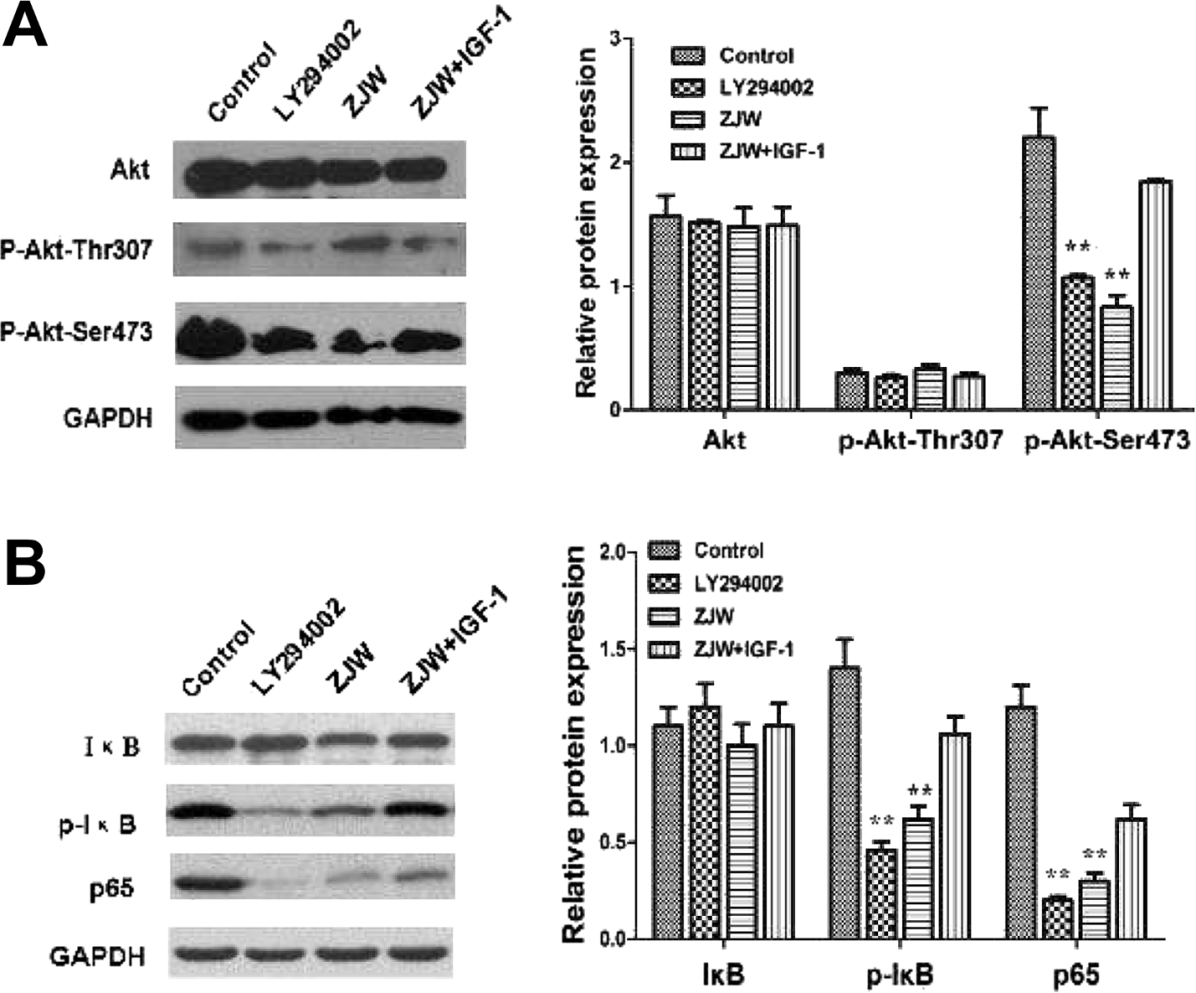
**ZJW suppresses P-gp mediated MDR by inhibiting activation of Akt (Ser473)/IκB phosphorylation (Ser473)** ***in vitro*** **. (A&B)** Western blotting assay was carried out to detect the level of Akt, phosphorylation of Akt (Thr307/Ser473), IκB, p65, and phosphorylation of IκB in HCT116/L-OHP cells treated with LY294002 (20 μM, 2 h), ZJW (50 μg/mL, 48 h), and a combination of ZJW (50 μg/mL, 48 h) and IGF-1 (100 ng/mL, 48 h). GAPDH was used to ensure equal loading of proteins in each lane. Data are means ± SD of values from triplicate experiments. **P < 0.01 *vs.* control group.

**Figure 5:**
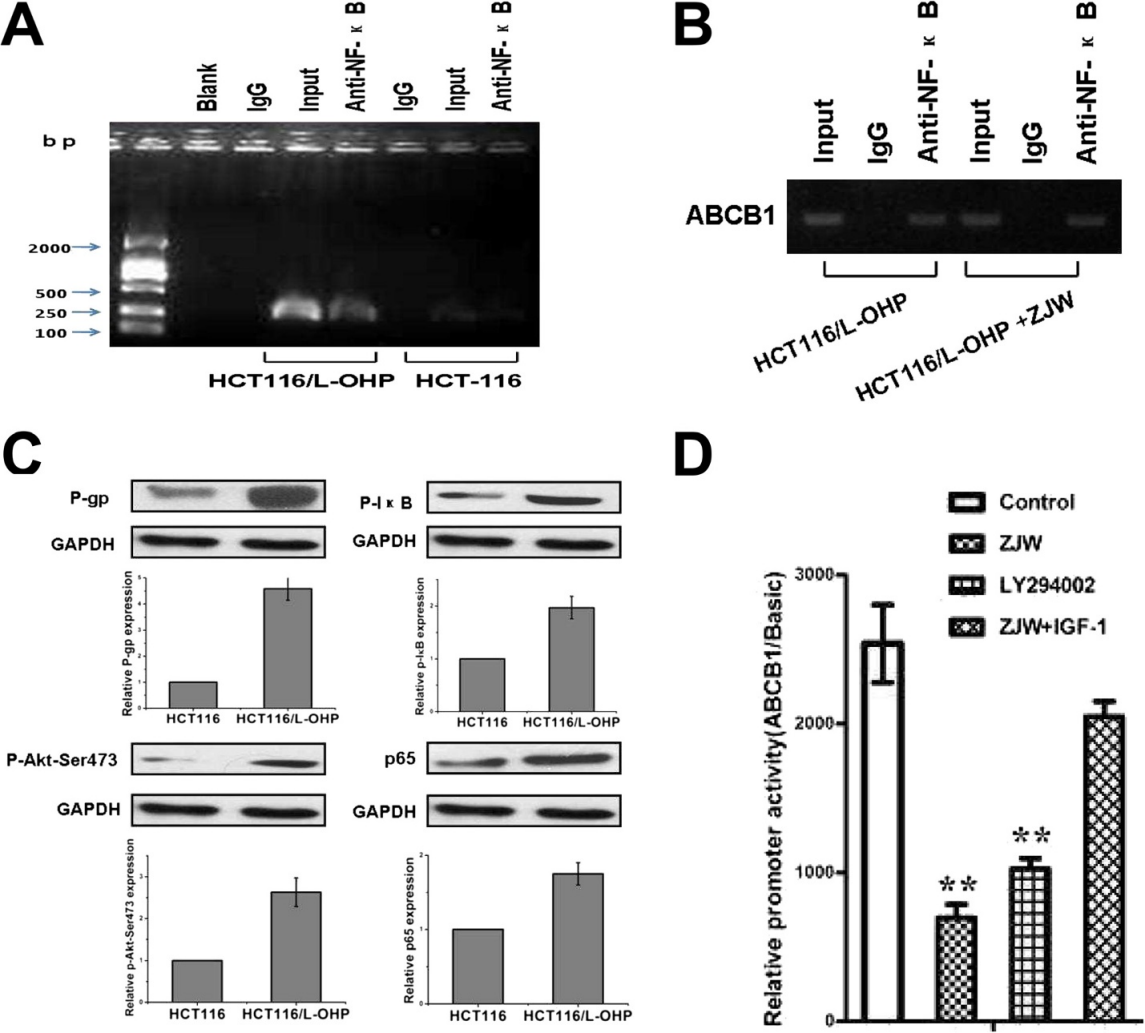
**ZJW suppresses P-gp mediated MDR by inhibiting activation of NF-κB pathway** ***in vitro*** **. (A)** ChIP analysis between NF-κB protein and ABCB1 gene. As a control, mouse monoclonal IgG was used. The blank group was the PCR results with no cDNA, and the input group was the cDNA from cell lysates without RIP procedure. **(B)** ChIP analysis was carried out to detect the effect of ABCB1 gene in HCT116/L-OHP cells treated with ZJW (50 μg/mL, 48 h). As a control, mouse monoclonal IgG was used. Input group was the cDNA from cell lysates without RIP procedure, and anti-NF-κB group was the cDNA from cell lysates after treatment with NF-κB antibody. **(C)** Western blotting assay was carried out to detect the level of P-gp, p-Akt (Ser473), p-IκB and p65 between HCT116 cell and HCT116/L-OHP cell. **(D)** The activity of the ABCB1 promoter in HCT116/L-OHP cells treated with LY294002 (20 μM, 2 h), ZJW (50 μg/mL, 48 h), and a combination of ZJW (50 μg/mL, 48 h) and IGF-1 (100 ng/mL, 48 h), was analyzed by a dual-luciferase assay kit. The results are the firefly luciferase/renilla luciferase ratio from different groups. Data are means ± standard deviation of values from triplicate experiments. **P < 0.01 *vs.* control group.

To determine whether the PI3K/Akt pathways are involved in the P-gp mediated drug-resistance phenotype in colorectal cancer, the expression of Akt and Akt phosphorylation (Thr307 and Ser473) were examined in HCT116/L-OHP cells by western blotting. Notably, ZJW or LY294002 decreased the expression of Akt phosphorylation (Ser473) in HCT116/L-OHP cells (Figure 4A), but did not significantly affect the expression of Akt or p-Akt at Thr307. Additionally, this inhibition was weakened after the addition IGF-1 (Figure 4A). These observations suggest that PI3K/Akt pathway activation could regulate the expression of P-gp, which is involved in controlling the drug-resistance phenotype.Evidence suggests that the PI3K/Akt pathway is involved in the development of chemoresistance, at least in part by the activation of NF-κB. In light of our results, we examined the effect of ZJW on NF-κB and phosphorylation of NF-κB in the cytoplasm and p65 levels in the nucleus. Similar to the effect on Akt and p-Akt, we observed a down-regulation of NF-κB phosphorylation in HCT116/L-OHP cells treated with ZJW or LY294002 (Figure 4B).Since the ABCB1 promoter is shown to bind with NF-κB, we hypothesized that there would be a down-regulation of ABCB1 promoter activity after treatment with ZJW. We found that the activity of the ABCB1 promoter was down-regulated after cells were treated with ZJW (Figure 5D). To further ascertain whether NF-κB protein could bind to the ABCB1 gene in HCT116/L-OHP cells, we tested NF-κB and ABCB1 by ChIP. ChIP assay confirmed that the NF-κB protein could bind to the ABCB1 gene promoter in HCT116/L-OHP cells, but not in HCT116 cells (Figure 5A). As presupposed, it indeed a down-regulation of ABCB1 mRNA expression after treatment with ZJW compared with control group (Figure 5B). Similar as the results in Figure 5A, the level of P-gp, p-Akt (Ser473), p-IκB and p65 were significantly increased in HCT116/L-OHP cell compared with HCT116 cell (Figure 5C). It indicated the difference between MDR cell and sensitive cell, which may be an important mechanism of drug-resistance phenotype. Therefore, ZJW was identified as a PI3K/Akt/NF-κB pathway inhibitor, which inhibits the phosphorylation of Akt and NF-κB and the binding of NF-κB and ABCB1 in MDR cell nuclei.

## Discussion

The aim of this study was to investigate whether the anti-drug-resistance effect of ZJW was via inhibition of PI3K/Akt/NF-κB signaling in colorectal drug-resistance cancer. Our previous studies demonstrated that ZJW could reverse the MDR phenotype by increasing the sensitivity of MDR cells to chemotherapeutic agents and inhibiting P-gp expression both *in vitro* and *in vivo*[1]. In the present study, we investigated the anti-cancer molecular mechanisms of ZJW ethanol extracts in signal pathways reversing drug-resistance.

In colorectal cancer, patients who do not respond to chemotherapy usually have a high expression of various ABC transporter pumps, which are located on the cytoplasmic side of the resistant cell membrane, resulting in an increased drug efflux [17]. In addition, the effectiveness of current chemotherapeutic agents is limited by drug resistance, thus the discovery of the mechanisms governing the cellular response to chemotherapy and develops a new strategy for treatments are priority [18]. Traditional Chinese prescriptions and formulae can be used as a therapy to effectively control cancer progression, improve quality of life, and prolong survival times instead of conventional chemotherapy [19–21].

Several studies have demonstrated that the PI3K/AKT pathway activates the NF-kB system, which could lead to increase the transcription of target genes such as ABCB1 [22], COX-2 [23], and p53 [24]. Therefore, a definitive relationship between the PI3K/AKT/NF-kB pathway and drug-resistance has yet to be established [25]. However, no report has examined the effect any drug on the reversal of drug-resistance via blockage of the PI3K/Akt/NF-kB signal pathway in colorectal cancer.

ZJW, a traditional Chinese herbal medicine, is viable and effective for inhibiting drug-resistance in lowest dosage of the IC_10_ to MDR cells [1]*.* In the present study, the concentration range of 50 μg/mL (IC_10_ to ZJW) was also used to help enhance the sensitivity of chemotherapeutic agents in the cell proliferation, apoptotic, but not in cell cycle. However, the mechanism of ZJW in the resistance to MDR cancer remains unknown. Earlier reports showed that evodiamine, a component of ZJW, could inhibit the PI3K/Akt pathway and targeted NF-κB in pancreatic cancer [26]. In our study, a remarkable activation of phosphorylation AKT and NF-κB was detected in HCT116/L-OHP cells, which also have up-regulation of P-gp. In addition, we showed that the levels of phosphorylation AKT and NF-κB in HCT116/L-OHP cells decreased after exposure to ZJW. However, the effects of ZJW in inhibiting of the level of P-gp, phosphorylation AKT, and NF-κB were weakened after treatment with the PI3K/Akt activator in HCT116/L-OHP cells. Therefore it is reasonable to believe that ZJW might play a suppressive role in the expression of P-gp via inhibition of the PI3K/Akt/NF-κB pathway.

These results are consistent with previous reports and may explain the effects of ZJW, including enhancing the inhibition rate of chemotherapeutic agents in a time-dependent manner, and helping chemotherapeutic drugs induce cell apoptosis in a dose-dependent manner. These results also agree with *in vivo* data that showed that ZJW was able to reduce tumor volume and induce cell apoptosis with the combination of chemotherapeutic agents [27]. Therefore, ZJW exhibits down-regulation of the expression of ABCB1/P-gp via inhibition of the PI3K/Akt/NF-κB pathway *in vitro*.

## Conclusions

**Figure 6:**
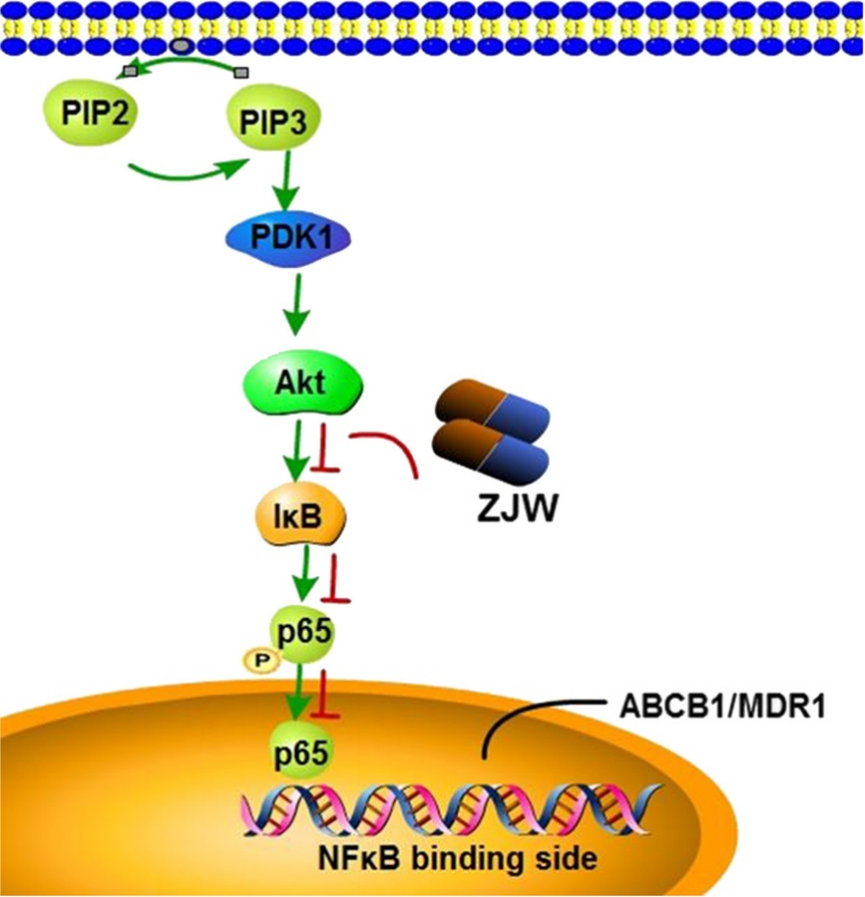
**Schematic summary of the inhibition mechanisms of ZJW on the PI3K/Akt/NF-κB signaling pathway.** Activation of PI3K/Akt/NF-κB pathway could reduce MDR phenotype. ZJW suppress phosphorylation of Akt (Ser473) and NF-κB.

In conclusion, based on our previous clinical and experimental studies, ZJW can be used as an inhibitor of chemoresistance in CRC. The present investigation has shown that the anti-MDR activity of ZJW can be partially attributed to the inhibition of the PI3K/Akt/NF-κB pathway and can modulate the binding of the ABCB1 promoter (Figure 6). Therefore, this study has provided a natural potent inhibitor of drug-resistance in human cancer. Compared with modern medicine, the combination of multiple herbs may yield better results in cancer treatment. The development of treatment with ZJW will be explored as a potential therapeutic strategy in human drug-resistance cancer.
